# Accurate Repositioning of Deep Brain Stimulation Electrodes May Preserve Clinical Efficacy

**DOI:** 10.7759/cureus.94031

**Published:** 2025-10-07

**Authors:** Robert Ziechmann, Katelyn Mann, Kevin Hines, Caio Matias, Ashwini Sharan, Chengyuan Wu

**Affiliations:** 1 Neurosurgery, Thomas Jefferson University Hospital, Philadelphia, USA

**Keywords:** deep brain stimulation, electrode repositioning, movement disorders, stereotaxic techniques, surgical technique

## Abstract

Background and objectives

Accurate placement of deep brain stimulation (DBS) electrodes is critical for therapeutic efficacy. Repositioning during the initial surgery is common and can achieve sub-millimeter accuracy. The impact of repositioning on clinical outcomes is not well studied, especially in an imaging-guided, imaging-verified (“asleep”) workflow. This study evaluates the effect of repositioning on clinical outcomes for people with Parkinson’s disease (PD), comparing clinical outcomes in cases where electrodes required repositioning to those where repositioning was not required.

Methods

We performed a retrospective analysis for PD patients undergoing DBS implantation between July 2018 and November 2024 at a single institution using an “asleep” technique. Electrodes were repositioned if radial error exceeded 1.5 mm or if adverse effects were anticipated based on anatomical electrode location. Radial error and final radial error were measured using the intraoperative O-arm. Clinical outcomes were assessed by percentage improvement in the Movement Disorders Society-Unified Parkinson’s Disease Rating Scale (MDS-UPDRS) part III score.

Results

Of 172 PD cases, 20 had complete preoperative and postoperative MDS-UPDRS III data, six of which required electrode repositioning. The revision group had a final radial error of 0.86 ± 0.29 mm, similar to 0.91 ± 0.43 mm in the non-revision group (p = 0.44). MDS-UPDRS III improvement was 38 ± 17% in the revision group and 40 ± 26% in the non-revision group (p = 0.44). Patient demographics (age, time since diagnosis, levodopa response) were similar between groups, except for a lower preoperative MDS-UPDRS part III score in the non-revision group (p = 0.04). The study was underpowered to formally detect non-inferiority.

Conclusion

This data suggests that repositioning sub-optimally placed DBS electrodes during initial asleep DBS surgery can achieve sub-millimeter accuracy and MDS-UPDRS part III improvement comparable to cases that did not require repositioning. However, our small sample size limits definitive conclusions, demonstrating a need for larger studies to confirm these findings that support intraoperative imaging-based verification of electrode location.

## Introduction

The long-term efficacy of deep brain stimulation (DBS) surgery depends on accurate placement in the therapeutic target. One series of 30 subthalamic nucleus (STN) DBS cases for Parkinson’s disease (PD) found that the only variable to predict outcome is placement of the active DBS contact within the STN [1]. A systematic review and meta-analysis of STN DBS for 1391 patients over 40 studies found that errors up to 1.86 mm did not have a negative effect on motor improvement in PD [2]. Further evidence that location matters comes from a series of 19 patients with poor initial response to DBS because of suboptimal electrode placement, 17 of whom improved after revision surgery to reposition the active contact into the originally intended therapeutic target [3].

The relationship between location and therapeutic effect has not been reported in the context of repositioning during the initial DBS surgery, an event that may occur in up to 19-39% of cases [4,5]. Repositioning during the initial surgery has been described in the traditional “awake” microelectrode recording (MER)-guided DBS surgery done under local anesthesia and guided by clinical exam using the Ben Gun (Alpha Omega Engineering, Nof HaGalil, Israel), a stereotactic apparatus that allows for the simultaneous placement of multiple MER electrodes in parallel channels spaced 2 mm apart [6,7]. This was found to result in no difference in clinical efficacy for DBS electrodes that required repositioning [7].

We recently reported our surgical technique for repositioning sub-optimally placed DBS electrodes during initial image-guided, image-verified surgery [8]. This technique involved creating a new trajectory for the repositioned electrode that assumes the factor causing electrode deviation on the first pass would occur again on the second pass. For example, if there was a radial posteromedial deviation of 1.5 mm from the original trajectory, we would plan a new target 1.5 mm anterolateral. We hypothesize that these deviations result from brain biomechanical properties [9], supported by our finding of the direction of radial error being identical from the first pass to the second pass in 71.74% of revisions. Our approach to repositioning resulted in a final radial error in cases requiring repositioning of 0.96 ± 0.38 mm, comparable to our previously reported accuracy of 0.9 mm [9]. While our initial report focused on electrode placement accuracy, here we shift our focus to clinical outcomes in the same cohort. We hypothesized that electrode repositioning within an “asleep” DBS workflow yields comparable benefit to that seen in “awake” workflow, provided accuracy remains within 2 mm. To test this hypothesis, we compared clinical outcomes in cases where electrodes required repositioning to those where repositioning was not required.

## Materials and methods

This was a retrospective analysis of patients who underwent DBS implantation for PD between July 2018 and November 2024 at the Thomas Jefferson University Hospital, Philadelphia, Pennsylvania, United States. The study was approved by the Office of Human Research Protection, Institutional Review Board of Thomas Jefferson University Hospital (approval number: 16D.321), which granted a waiver of informed consent due to the retrospective nature of the analysis. All DBS surgeries were performed by one of four fellowship-trained functional neurosurgeons. Patients who required lead repositioning during their initial surgery were studied in further detail.

Sample size calculation

To detect a difference in clinical outcomes, we performed a power calculation based on the literature. Improvement in the Movement Disorders Society-Sponsored Unified Parkinson's Disease Rating Scale (MDS-UPDRS) part III score [10] has been reported as approximately 40% [11-17] with a standard deviation of 10% [11,12,15]; and the meaningful clinical difference as 15% or 5 points [18, 19]. For a t-test with a power of 80% demonstration of non-inferiority would require at least 23 patients in each group

Surgical technique

All patients underwent DBS surgery under general anesthesia with endotracheal intubation in an image-guided, image-verified workflow, as we have previously described [11]. The head is fixed to a stereotactic robot via a Leksell frame, and an O-arm scan is used for registration. After an incision is made, a high-speed drill is used to create a 3.2 mm craniostomy. The dura and pia are opened with low-power monopolar cautery placed through the robot arm in the trajectory of the electrode. Based on surgeon preference, the electrode is placed with or without a cannula. The electrode is secured with a titanium plate covered with a silastic sheath.

An intraoperative O-arm scan is obtained after the electrode (or both electrodes in a bilateral case) is placed and secured, and the scan is coregistered with preoperative imaging on the planning station. Electrodes are generally repositioned if the target error exceeds 1.5 mm and/or if adverse effects are anticipated based on the anatomical location of the electrode. For example, if at least half of the electrodes appeared to be in the internal capsule on the O-arm scan, it was repositioned as a rule.

The second pass of the electrode via the adjusted trajectory was generally performed via a new, separate entry point craniostomy, repeating the same aforementioned steps while accounting for the direction and magnitude of deviation from the intended trajectory [8]. If more than one pass is required, the steps are repeated. Once the electrode position is acceptable, it is secured with the titanium mini-plate.

Imaging analysis

Final radial error was measured on the neuroinspire™ surgical planning software (Renishaw plc, Wotton-under-Edge, United Kingdom) based on the intraoperative O-arm. We measured the final radial error from the center of the distal-most contact of the implanted electrode on the distal-most slice, where the artifact is clearly visible, to the center of the planned trajectory.

Clinical outcomes and data analysis

Basic demographic information was recorded for patients undergoing repositioning during their initial DBS surgery. Only patients undergoing de novo DBS implant were included in our analysis to avoid confounding from prior DBS therapy and potential habituation (adaptive neurologic changes in the circuit targeted). The primary clinical outcome of interest was the percentage improvement in the UPDRS III score comparing the on-medication score from within six months before the DBS surgery to the on-medication, on-stimulation score within the first year of DBS stimulation. Also recorded was the accuracy in the form of the final radial error. Clinical outcomes data within six months before DBS surgery and within one year afterward were available for most patients; however, formal outcome scale measures with MDS-UPDRS III were only available for a few. We also did an analysis on subsets of cases based on target, STN, or globus pallidus internus (GPi). Revision and non-revision groups were compared using an independent two-tailed t-test for continuous variables and using a Fisher’s exact test for categorical variables.

## Results

There were 172 patients with PD who underwent DBS, of whom 38 required repositioning. Unfortunately, only 20 patients had a MDS-UPDRS III score clearly documented both within six months before surgery and one year after surgery. Of the 20 PD patients with both UPDRS III scores available, six required repositioning during their initial surgery. As such, we did not meet sufficient numbers by our power analysis to detect non-inferiority; however, given that there is currently no data published on clinical outcomes in this context, we present this data as a preliminary analysis as we continue to gather data.

Patient demographics are listed in Table 1. Preoperative MDS-UPDRS was 11.5 points lower on average in the group that did not require repositioning (p = 0.04); however, levodopa response was no different (p = 0.43).

**Table 1:** Demographic and clinical information of included patients Age, time since PD diagnosis, preoperative MDS-UPDRS part III [10], and MDS-UPDRS levodopa response were compared with a t-test. Sex (M/F ratio) was compared using Fisher's exact test. The MDS-UPDRS was used with permission on a per-study license from the International Parkinson and Movement Disorder Society. MDS-UPDRS: Movement Disorders Society-Unified Parkinson’s Disease Rating Scale

	Non-Revision	Revision	Test Statistic	p-value
Age (years)	62 ± 11	60 ± 8.9	t = -0.36	P = 0.37
Sex (M/F ratio)	8:6	5:1	N/A	P = 0.35
Time since PD diagnosis (years)	6.7 ± 3.4	5 ± 3.2	t = -1.10	P = 0.14
Preoperative MDS-UPDRS	36 ± 13.28	47.5 ± 12.47	t = 2.34	P = 0.04
MDS-UPDRS levodopa response	38% ± 20%	34% ± 8.7%	t = -0.65	P = 0.43

Outcomes are listed in Table 2. The average improvement in the MDS-UPDRS part III score after DBS implantation was 39.4% ± 23.6% across our cohort. This was similar between patients who required repositioning versus those who did not (p = 0.44). The average final radial error for all cases was 0.92 ± 0.40 mm. This was similar between patients who required repositioning versus those who did not (p = 0.44).

**Table 2:** Comparison of improvement in MDS-UPDRS patients who required revision during their initial surgery and those who did not The MDS-UPDRS [10] was used with permission on a per-study license from the International Parkinson and Movement Disorder Society. MDS-UPDRS: Movement Disorders Society-Unified Parkinson’s Disease Rating Scale

Parameters	Non-Revision	Revision
MDS-UPDRS part III improvement	40% ± 26%	38% ± 17%
Final Radial Error	0.91 ± 0.43 mm	0.86 ± 0.29 mm

For patients with STN electrodes, MDS-UPDRS III improvement was 33% ± 11% in the three patients who required repositioning compared to 48% ± 30% in the four patients who did not (p = 0.42). Final radial error was 0.84 ± 0.30 mm in the patients who required repositioning versus 1.08 ± 0.41 mm in the patients who did not (p = .41). For patients with GPi electrodes, MDS-UDPRS III improvement was 44% ± 22% in those who required repositioning (n = 3) versus 37% ± 25% in those (n = 10) who did not (p = 0.70). Final radial error was 0.87 ± 0.36 mm for the patients who required repositioning compared to 0.89 ± 0.43 mm for the patients who did not (p = 0.89).

## Discussion

This preliminary data suggests that repositioning sub-optimally placed DBS electrodes during asleep DBS surgery with our technique results in comparable MDS-UPDRS III improvement (38% ± 17%) to non-revision cases (40% ± 26%) when there is sub-millimeter accuracy (0.86 ± 0.29 mm).

Our findings are consistent with previous work showing preserved clinical efficacy for awake DBS surgeries requiring repositioning using the Ben Gun, reporting no statistical difference in benefit from neurostimulation in those who required repositioning and those who did not [7]. Our study suggests a similar outcome for the asleep DBS workflow, where a final sub-millimeter radial error in both the revision and non-revision groups may lead to similar clinical outcomes in those who require repositioning and those who do not. Our clinical outcomes with MDS-UPDRS improvement of 39.4% ± 23.6% across our cohort are also in line with those reported in the literature for MDS-UPDRS improvement of 40% ± 10% [11-17]. Our findings are particularly relevant given the increasing adoption of asleep DBS techniques, which rely on imaging rather than intraoperative clinical improvement or MER physiology [20,21]. Giving priority to the importance of anatomy in clinical outcomes is also important as the indications for DBS grow to include targets for which MER physiology and intraoperative clinical testing are either not feasible or do not provide definitive information, such as the thalamic targets for epilepsy.

Previous work has shown that electrode placement within 2 mm of the therapeutic target is an important threshold for optimal clinical outcome. It has been shown previously that accurate placement of the active DBS contact within the STN was the primary predictor of motor improvement in PD [1]. Radial error up to 1.86 mm does not significantly decrease motor outcomes [2]. Our revision group’s final radial error (0.86 ± 0.29 mm) falls well within this threshold. We also provide preliminary evidence for this effect in patients with GPi electrodes, not just STN.

While prior work has shown that repositioning sub-optimal electrodes after the initial DBS surgery (in a separate revision surgery) restored clinical efficacy in 17 of 19 patients with sub-optimal placements [3], our study suggests that this concept extends to intraoperative repositioning. Verifying electrode location with intraoperative imaging before leaving the operating room and repositioning as needed may reduce the need for additional surgeries and their associated risks, such as hemorrhage [4].

Limitations

Our study has several limitations. The foremost limitation is the inadequate sample size, which is not powered to detect non-inferiority. Our analysis focused on motor outcomes (MDS-UPDRS III) and did not assess non-motor outcomes or long-term efficacy beyond one year, which could differ between revision and non-revision groups. Finally, our approach is specific to asleep DBS with a minimally invasive workflow, and its applicability to awake DBS is unclear.

Future directions

Larger, adequately powered studies are needed to validate these preliminary findings across PD as well as other DBS indications. Advanced imaging, such as magnetic resonance elastography, could further define biomechanical factors driving electrode deviations, which, when accounted for, could potentially reduce the need for repositioning. Minimizing additional electrode passes remains critical to reducing hemorrhage risk.

## Conclusions

This underpowered, preliminary study suggests that intraoperative repositioning of sub-optimally placed DBS electrodes in asleep DBS surgery may achieve high accuracy (0.86 ± 0.29 mm) and comparable MDS-UPDRS III improvement in non-revision cases in PD patients. The systematic approach, accounting for reproducible biomechanical deviations, offers a promising framework for asleep DBS workflows. However, the small sample size precludes definitive conclusions, underscoring the need for larger studies to establish clinical equivalence and generalizability.
